# Impact of PEEK implant surface design on postoperative complications in cranioplasty: a retrospective review

**DOI:** 10.1186/s41016-025-00417-3

**Published:** 2025-11-21

**Authors:** Carmen A. Zavala, Laura Zima, Subhiksha Srinivasan, Sanjay V. Neerukonda, Mark J. Dannenbaum

**Affiliations:** 1 McGovern Medical School, University of Texas Health Science Center at Houston, 6431 Fannin Street, Houston, TX 77030 USA; 2 Vivian L. Smith Department of Neurosurgery, University of Texas Health Science Center at Houston, Houston, TX USA

**Keywords:** Polyetheretherketone, PEEK, Cranioplasty, Craniectomy, Computer-designed cranioplasty implants

## Abstract

**Background:**

The aim of this study was to evaluate and compare complication rates and clinical outcomes associated with smooth and perforated polyetheretherketone (PEEK) implants used in cranioplasty.

**Methods:**

A retrospective analysis of 94 patients who underwent cranioplasty with either smooth (*n* = 45) or perforated (*n* = 49) PEEK implants over a five-year period was conducted. Patient demographics, comorbidities, reasons for initial craniectomy, time interval between craniectomy and cranioplasty, postoperative complications, hospital stays, and rates of revision surgeries were analyzed. Multivariate logistic regression was used to control for confounding factors.

**Results:**

No statistically significant differences were observed in demographic characteristics, reasons for initial craniectomy, or median time to cranioplasty between groups. Complication rates including wound complications, infections, ventriculoperitoneal (VP) shunt placements, significant fluid collections, return to surgery, and implant removals were comparable between groups, though trends suggested potential increases in wound complications (17.8% vs. 8.2%, *p* = 0.11) and infections (17.8% vs. 8.2%, *p* = 0.22) in the smooth implant group. Interaction analysis indicated a significant reduction in significant fluid collections with smooth implants in trauma patients (*p* = 0.045). Importantly, a rare and previously unreported case of malignant cerebral edema following smooth PEEK implant placement was documented.

**Conclusions:**

Although no statistically significant differences were found, the identified trends toward increased complications with smooth implants and the novel finding of malignant cerebral edema highlight the importance of implant surface characteristics. Further prospective randomized studies are needed to clarify these preliminary observations and guide clinical decision-making in cranioplasty procedures.

## Highlights

• PEEK cranioplasty implants are custom designed with either smooth or perforated surfaces, the choice of which is typically based on surgeon preference.

• No significant differences were found between smooth and perforated PEEK implants, but trends suggest higher rates of wound complications and infections with smooth implants.

• This study presents the first documented case of malignant cerebral edema associated with a smooth PEEK cranioplasty implant.

• Surface architecture of PEEK implants, particularly porosity and perforation, may impact osseointegration and clinical outcomes, highlighting an area ripe for future research.

## Background

A cranioplasty is a common neurosurgical procedure performed to restore skull defects and improve functional and cosmetic outcomes following craniectomy. Standard procedure is replacing the skull defect with either the patient’s autologous bone or synthetic implants. While autologous bone flaps are the gold standard, they carry a high complication rate (up to 50%) [1]. The most common synthetic materials used in cases of non-autologous cranioplasty include titanium mesh (TM), hydroxyapatite, polymethyl methacrylate (PMMA), or polyetheretherketone (PEEK). Computer-aided design and manufacturing is utilized to fit patient-specific defects in order to decrease complications and increase cosmetic outcomes. Generally, the type of implant material chosen is driven by surgeon preference and cost of material. Recent work has shown that autologous flaps have higher complication rates than synthetic implants [2]. Because of this, neurosurgeons are more commonly choosing synthetic implants, however open questions remain concerning which material has the best post-surgical outcomes.

PEEK implants are a common choice for cranioplasty because this material has similar tensile properties to bone, is thermodynamically stable, demonstrates high resistance to radiation, and is also radiolucent and compatible with MRI and ultrasound [3]. In addition, PEEK implants can be 3D printed, allowing for modifications [4]. Other aspects that result in surgeon preference of PEEK implants are ease and time efficiency [4]. PEEK implants are also chosen to reduce rates of revision cranioplasty [4]. Although studies show that PEEK implants have a higher success rate and lower complication rate than autologous bone grafts, their complication rates remain variable [2, 5]. Some complications that have been observed in prior literature with PEEK implants include infection, postoperative hematoma, cerebrospinal fluid leak, and wound complications [6]. While this material has been used extensively in other fields like orthopedics, maxillofacial, and cardiac surgery, its efficacy remains to be more robustly explored with respect to cranioplasties [7]. PEEK is chemically inert and consequently poses a challenge in osseointegration compared to other materials [7]. Many attempts to modify the surface of PEEK implants have been attempted to promote osseointegration and decrease microbial susceptibility, such as bioceramic and titanium coatings and structural modifications, such as creation of a porous surface [7]. To date, these implant properties have not been compared in cranioplasties.

**Fig. 1:**
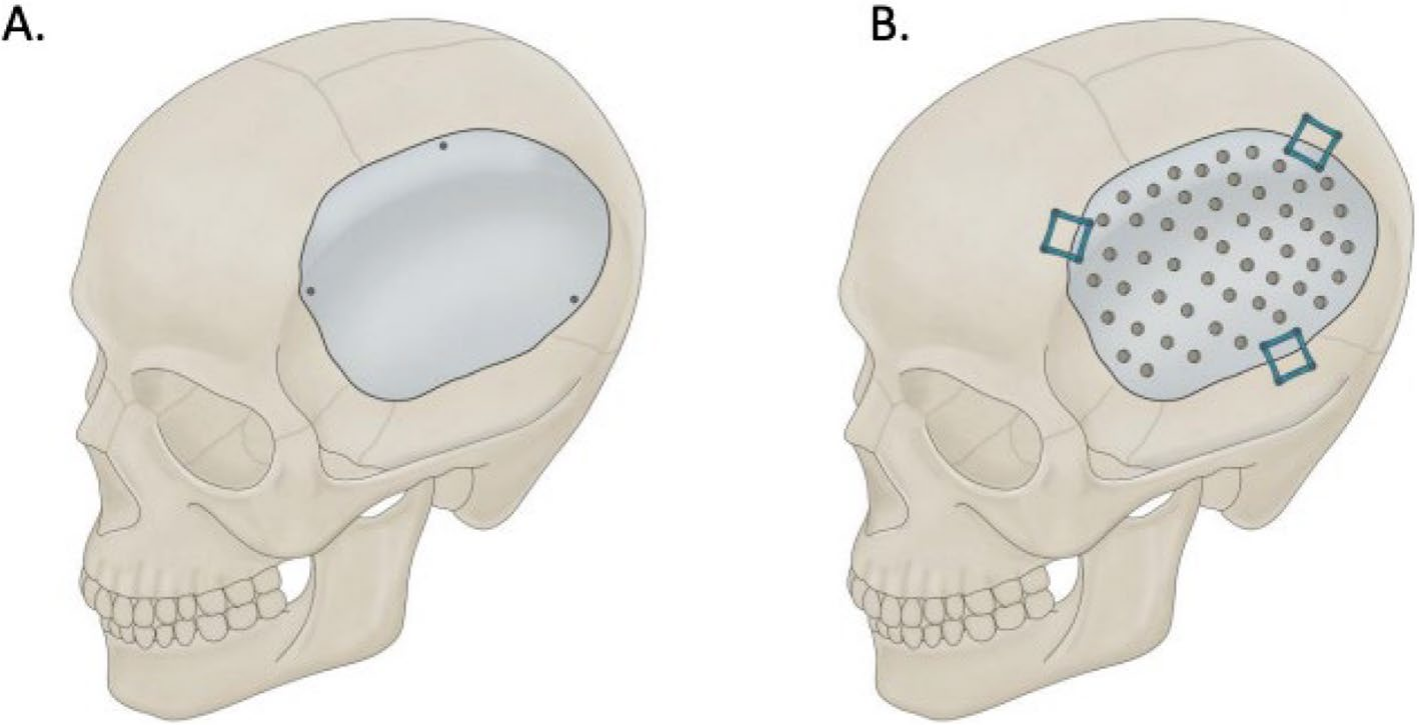
PEEK Cranioplasty Subtypes. **A** Smooth model. **B** Perforated model. Medical Illustration by Robert Suazo UTHealth Houston Neurosciences

The PEEK implants typically used in cranioplasties are custom ordered with either a smooth solid surface (Fig. 1, A) or perforated with 2–3 mm suture/drainage holes (Fig. 1, B). However, material selection is currently based typically on surgeon preference, and so further exploration of statistically meaningful differences between these, in terms of complication profiles, could provide additional information relevant for surgical decisions. Given that prior studies have demonstrated the importance of PEEK implant surface structure, we explore if there are significant differences in postoperative outcomes between the smooth and perforated PEEK implants used in cranioplasty. While these perforated holes can serve to tack up the dura, multiple studies have shown that the surgical practice of dural tenting does not impact post-surgical outcomes so this paper will not focus on surgical technique, but rather on the implant surface type. In our study, CNC machine-milled medical-grade PEEK implants were used, which had a 3 mm body and 4 mm fixation area. In perforated implants, profusion holes were 2 mm in diameter. The holes were even spaced 10 mm apart center to center, with no holes within 15 mm of implant edge. We made sure to collect data based on complications observed in prior literature, including infection, stroke and fluid collections [8].We identify across material types the hospital stay duration, infection rates, post-operative fluid collections, and need for surgical revision. In brief, we leverage additional data and direct statistical comparisons in the service of contributing to evidence-based selection of PEEK implant subtype.

## Methods

### Study design and cohort

**Fig. 2:**
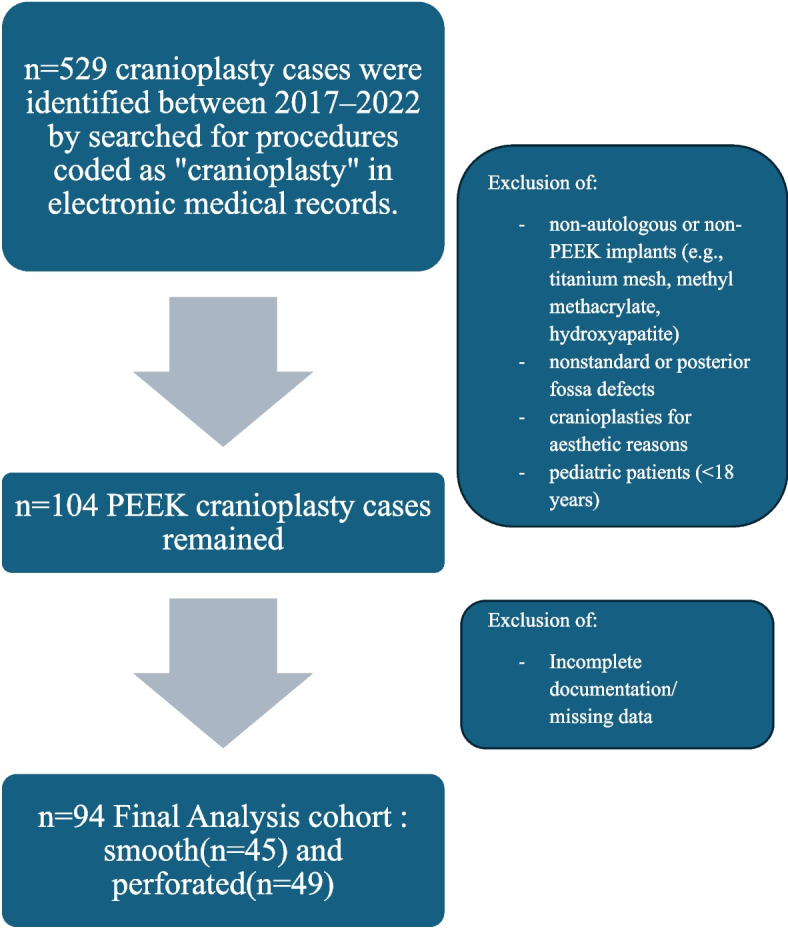
Methodology Flow Diagram

All cranioplasty procedures were queried at our institution from 2017–2022. A total of 529 procedures coded as “cranioplasty” were obtained from the electronic medical records(EMR) system. Autologous cranioplasties were excluded and any cranioplasty for a defect other than a standard decompressive hemicraniectomy defect was excluded. Additionally, patients were excluded if they were pediatric (defined as < 18 years old). After applying exclusion criteria, 104 procedures remained. Of these, 10 procedures had incomplete or missing data, and were therefore not included in the final analysis cohort. A flow diagram detailing this process is provided in Fig. 2.

### Data collection

Patient data was extracted including original injury, surgery, type of implant (smooth vs perforated), and post-operative course including any complications related to the cranioplasty including future surgeries. Data was then de-identified for analysis and reporting.

In relation to comorbidities, patient data regarding the presence of four comorbidities were identified: hypertension, diabetes, coronary artery disease, and coagulopathy. Trauma, intracranial hemorrhage/hemorrhagic stroke, ischemic stroke, aneurysm, tumor resection, and abscess were identified as the surgical indication for craniotomy/craniectomy in this cohort. Due to small sample sizes, trauma and intracranial hemorrhage/hemorrhagic stroke (ICH) were evaluated separately, while ischemic stroke, aneurysm, tumor resection and abscess were grouped into an “other” indications category to allow for more accurate statistical analysis. Significant fluid collections were diagnosed based on post-operative CT scan by neurosurgeons and radiologists. Collections were considered significant if additional imaging was ordered for monitoring and/or if surgical revision was required. Wound complications consisted of wound infections, dehiscence, and other complications that required clinical management or surgical revision. Any reason for surgical re-intervention, including infection or fluid collections, was recorded as “return to surgery.” Prolonged hospital stay was defined as ≥ 5 days due to prior literature referencing median cranioplasty hospital stay as 4.6 days [9]. No patients were on anticoagulation therapy at the time of cranioplasty.

### Statistical analysis

Python 3.9.18 and R 4.4.3 were used to calculate statistics. Univariate statistics were performed using Fisher’s exact test and Mann Whitney’s U test. Additionally, a multivariate analysis was performed using Firth’s logistical regression analysis due to small sample sizes. A post-hoc power analysis was performed to contextualize the statistics in the realm of small sample sizes. The analysis was performed using a two-proportion approximation for all binary outcomes using established statistical methodology. Therefore, the post-hoc power analysis only provides approximations since Fisher’s exact test was used for univariate calculations.

Separate multivariate regression models were constructed for each of the following outcome variables: significant fluid collections on CT, wound complications, postoperative infection, return to surgery, VP shunt placement, and PEEK implant removal. These models were adjusted for the patient characteristic variables, including age, time to cranioplasty, reason for craniectomy, and number of comorbidities. Models that included interactions between type of cranioplasty (smooth versus perforated) and reason for cranioplasty were also performed. All multivariate analysis was conducted using Firth’s logistical regression.

## Results

A total of 94 patients who underwent cranioplasty with either smooth (*n* = 45) or perforated (*n* = 49) implants were included in the analysis. Demographic data (Table 1) demonstrated comparable patient characteristics between both groups, with no statistically significant differences identified in age (median age 40.9 vs. 38.8 years, *p* = 0.98), prevalence of comorbidities such as hypertension, diabetes mellitus, coronary artery disease, cancer, and coagulopathy, or the primary indication for craniectomy. Trauma was the most common reason for craniectomy in both groups (68.9% smooth vs. 53.1% perforated, *p* = 0.14), followed by intracranial hemorrhage (22.2% vs. 20.4%, *p* = 1.00). Median time to cranioplasty was similar between groups (8 months smooth vs. 8.2 months perforated, *p* = 0.97).

**Table 1:** Patient Demographics per Implant Type

	**Smooth (*n* = 45)**	**Perforated (*n* = 49)**	***P* value**
**Age (median (IQR))**	40.9 (33.0–53.3) years	38.8 (27.7–61.0) years	0.98
**Comorbidities**			0.98
Hypertension	16/45 (35.6%)	14/49 (28.6%)	0.51
Diabetes Mellitus	4/45 (8.9%)	6/49 (12.2%)	0.74
Coronary Artery Disease	1/45 (2.2%)	2/49 (4.1%)	1.00
Cancer	1/45(2.2%)	1/49(2.2%)	1.00
Coagulopathy	6/45 (13.3%)	8/49 (16.3%)	0.78
**Reason for Craniotomy**			
Trauma	31/45 (68.9%)	26/49 (53.1%)	0.14
Intracranial Hemorrhage	10/45 (22.2%)	10/49 (20.4%)	1.0
Ischemic Stroke	1/45 (2.2%)	2/49 (4.1%)	1.0
Aneurysm	1/45 (2.2%)	3/49 (6.1%)	0.62
Tumor Resection	1/45 (2.2%)	5/49 (10.2%)	0.21
Abscess	1/45 (2.2%)	3/49 (6.1%)	0.62
**Time to Cranioplasty (median (IQR))**	8 (4.4–13.3) months	8.2 (4.6–16.1) months	0.97

Values are presented as median with interquartile range (IQR) or as a percentage (%) based on the variable type

Time to cranioplasty = time interval between craniectomy and subsequent cranioplasty

Fisher’s exact test used to calculate p values for univariate analysis for categorical and binary variables

Mann–Whitney U test used to calculate p values for univariate analysis for continuous variables

All *p* values are exact

Univariate analysis (Table 2) revealed no significant differences between smooth and perforated implants regarding complications. Post-operative CT findings revealed significant fluid collections in 2.2% of patients with smooth implants compared to 10.2% with perforated implants (*p* = 0.210). Rates of wound complications (17.8% vs. 8.2%, *p* = 0.11), postoperative infections (17.8% vs. 8.2%, *p* = 0.22), return to surgery (28.9% vs. 20.4%, *p* = 0.47), ventriculoperitoneal (VP) shunt placement (13.3% vs. 6.1%, *p* = 0.30), and implant removal (11.1% vs. 12.2%, *p* = 1.00) were statistically comparable. Median hospital stay was 3 days for smooth implants versus 2 days for perforated implants (*p* = 0.19), with no difference in prolonged hospital stays (≥ 5 days; 20% vs. 18.4%, *p* = 1.00).

**Table 2:** Outcomes and Complications per Implant Type

Complications and Outcomes	Smooth (*n* = 45)	Perforated (*n* = 49)	*p* value	Post-hoc power analysis(%)
Significant fluid collections on CT	4	7	0.53	12.9
Wound Complications	8	4	0.22	28.7
Postoperative infection	8	3	0.11	41.9
Return to Surgery	13	10	0.47	15.9
VP shunt placement	6	3	0.30	22.1
PEEK implant removal	5	6	1.00	5.3
Hospital Stay (median (IQR))	3.0 (2.0–4.0) days	2.0 (2.0–3.0) days	0.19	
Number of Patients with Prolonged Hospital Stay (≥ 5 days)	9	9	1.00	5.5

Values are presented as n or number of patients who had the complications/outcomes presented in the table

Prolonged stay = ≥ 5 days after cranioplasty

Trauma and intracranial hemorrhage (ICH) were analyzed separately, while ischemic stroke, aneurysm, tumor resection, and abscess were grouped as “other” indications

Significant fluid collections were classified as such on postoperative CT when additional imaging or surgical revision was required

Wound complications included infection, dehiscence, or other issues requiring clinical or surgical management

“Return to surgery” represents reoperation for any reason

Fisher’s exact test used to calculate p values for univariate analysis for categorical and binary variables

Mann–Whitney U test used to calculate p values for univariate analysis for continuous variables

Post-hoc power analysis calculated for two-proportion comparison as an approximation (α = 0.05)

All *p* values are exact

Multivariate logistic regression analysis adjusted for patient age, time to cranioplasty, reason for craniectomy, and number of comorbidities (Table 3) did not demonstrate significant associations between implant type and adverse outcomes. Smooth implants trended toward increased wound complications (*p* = 0.123), postoperative infections (*p* = 0.170), and VP shunt placement (*p* = 0.192), though these findings did not reach statistical significance. Craniotomies for intracranial hemorrhage were significantly associated with increased VP shunt placement (*p* = 0.021). Increased age was significantly associated with prolonged hospital stay (*p* = 0.050) and showed a trend toward decreased VP shunt placement (*p* = 0.086). Greater time to cranioplasty showed trends toward increased VP shunt placement (*p* = 0.125) and wound complications (*p* = 0.164). A greater number of comorbidities showed trends toward increased prolonged hospital stay (*p* = 0.066) and return to surgery (*p* = 0.197). Interaction analysis revealed smooth cranioplasties significantly reduced significant fluid collections in patients who underwent craniotomies for trauma (*p* = 0.045).

**Table 3:** Significance and Trends of Non-Interaction Multivariate Analysis

Model	Variable	Coefficient (coef)	Standard Error	Lower 0.95 CI	Upper 0.95 CI	Chi-sq	*p*-value
Wound Complications	Type of Cranioplasty (Smooth)	1.024	0.649	−0.269	2.528	2.377	0.12
	Time to Cranioplasty	0.0150	0.00996	0.00712	0.0355	1.935	0.16
Infection	Type of Cranioplasty (Smooth)	0.856	0.608	−0.361	2.206	1.882	0.17
Return to Surgery	Comorbidity Count	0.422	0.320	−0.225	1.076	1.664	0.20
VP shunt placement	Type of Cranioplasty (Smooth)	0.930	0.691	−0.461	2.505	1.699	0.19
	Age	−0.0468	0.0267	−0.111	0.00619	2.952	0.09
	Time to Cranioplasty	0.0210	0.0116	−0.00732	0.0446	2.356	0.13
	Reason for Craniotomy (ICH)	2.114	0.884	0.326	4.074	5.364	**0.02**
Implant Removal	Reason for Craniotomy (ICH)	−1.611	1.373	−6.514	0.683	1.661	0.20
Prolonged Hospital Stay	Age	−0.0390	0.0204	−0.0846	1.22 × 10^–5^	3.839	**0.05**
	Comorbidity Count	0.684	0.366	−0.0450	1.476	3.383	0.07

Multivariate logistic regression performed without interaction terms using Firth’s logistic regression

*CI* confidence interval, *coef* coefficient, *ICH* intracranial hemorrhage

Significant *p* values bolded

Overall, 11 (12%) patients experienced wound complications or infections, with eight requiring implant removal (five smooth and three perforated). The predominant pathogens isolated were Staphylococcus aureus (7 cases) and Propionibacterium acnes (1 case). Trauma (7) and ischemic stroke (1) were the original indications for craniectomy in this group. One patient with a smooth PEEK implant had a rare complication of malignant cerebral edema.

## Discussion

PEEK cranioplasty implants are gaining popularity amongst neurosurgeons due to their tensile properties, thermodynamic stability, high resistance to radiation, radiolucency, and MRI and ultrasound compatibility. Compared to the gold standard of autologous bone, PEEK implants show fever complications, shorter hospital stay, and operation time [3, 8, 9]. However, PEEK implants have also been shown to have comparable hospital stay in comparison to autologous bone [10]. Therefore, although the implant has many benefits, post-operative complications still require investigation. The goal of this study was to evaluate the effect of PEEK implant subtype (smooth or perforated with suture/drainage holes) on post-operative complications.

We identified 94 patients who underwent cranioplasty with PEEK implants at a single institution over a five-year period. This study revealed no statistically significant differences in clinical outcomes or complication rates between smooth and perforated PEEK implants. However, notable trends emerged, indicating a potential increase in wound complications (*p* = 0.12), postoperative infections (*p* = 0.17), and VP shunt placements (*p* = 0.19) associated with smooth implants, though these did not reach statistical significance. Additionally, craniotomies for intracranial hemorrhage were significantly associated with higher rates of subsequent VP shunt placement (*p* = 0.02). Interaction analysis demonstrated a significant reduction in significant fluid collections for smooth implants in trauma cases (*p* = 0.05), emphasizing the potential importance of implant selection tailored to patient-specific clinical scenarios.

Our analysis did not yield statistically significant differences between smooth and perforated implants. However, it is important to consider the trends towards significance in the study due to the small sample size. The post-hoc power analysis also supports the impact of small sample size on statistical analysis. Yet, the statistically insignificant data further supports more clinical investigation.

PEEK is chemically inert and has low bioreactivity, leading to the formation of fibrous layer around the implant and poor integration with the host bone [11]. There has been much exploration into modifying the traditionally smooth PEEK implant to enhance integration. Studies show that smooth PEEK implants result in less osseointegration compared to those with other structural modifications, including porous surfaces and bioceramic/titanium coating [7, 12–15]. Osseointegration may also play a role in complication profiles. However, this impact has been sparsely described in literature when comparing smooth versus perforated PEEK implants. In our study, there were no statistically significant differences in patient outcomes. Therefore, the literature on implant surface structure impacting osseointegration and its eventual impact on outcomes warrants additional investigation in the clinical setting.

Our results as reported within the context of an ongoing discussion concerning complication profiles in cranioplasties. Current literature indicates that older patients and those with longer interval between craniectomy and cranioplasty typically experienced increased cranioplasty complications and odds of cranioplasty removal [16]. However, there are other studies that showed earlier cranioplasty (within 90 days) had higher odds of hydrocephalus with no difference in overall complications, infections, or reoperations [17]. In the present study, there was no association of length of time to cranioplasty with infection rate or wound revision. The most optimal timing of cranioplasty remains debated in the literature.

**Fig. 3:**
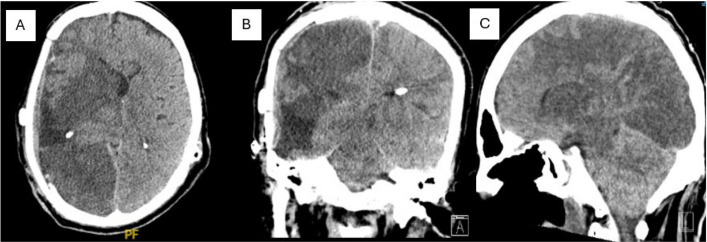
MRI of Malignant Cerebral Edema. **A**. Axial CT **B**. Coronal CT **C**. Sagittal CT

Malignant cerebral edema (MCE) is a rare (2.2% incidence), often fatal complication following cranioplasty, characterized by rapid neurological decline and widespread bilateral cerebral edema [18]. MCE has been previously reported with autologous, titanium, and methyl methacrylate implants. However, to our knowledge, a patient in our series highlights the first documented occurrence specifically involving a smooth PEEK implant. The patient was a 60-year-old male who previously underwent complete resection of a left sphenoid wing meningioma, subsequently complicated by infection, bone flap removal, and hydrocephalus requiring VPS placement. Two months later, after infection treatment, he received a smooth PEEK cranioplasty. Postoperatively, the patient suffered a seizure and rapid neurological deterioration ultimately leading to brain death. Imaging confirmed diffuse malignant cerebral edema and strokes in the brainstem and cerebellum (Fig. 3). Malignant cerebral edema after cranioplasty is a devastating and rare complication, though likely under-reported. It is thought to be due to dysregulation of cerebral blood flow after cranioplasty or negative pressure drainage [19]. Patients with sinking flap/trephination syndrome, traumatic etiology of original craniectomy, and cranioplasties performed early (less than three months) are thought to be higher risk for development of this syndrome [20]. There has been no association found between this devastating complication and type of implant. While it is clinically important to discuss this rare outcome, we have described only one case of MCE. This is a limitation of our rare case presentation, as conclusions cannot be drawn from a single patient outcome. It is important to interpret the case in this context. The discussion of this case was, rather, included to present an interesting, rare patient outcome.

Our study is limited by the fact that it is retrospective in nature and was completed at a single institution. Additionally, while attending was not a significant factor in revision rates, there was a small subset of attendings who preferred the smooth implant, three, vs a larger group that preferred the perforated (14). While this is certainly a confounder, it is somewhat unavoidable as attendings have specific preferences by nature. Future directions include a clinical randomized trial of smooth vs perforated implants which would control for factors such as attending preference. In addition, there were multiple other confounders that we could not account for both due to the small sample size limiting the number of variables that could reasonably be analyzed as well as the retrospective study design. Some of these critical clinically relevant factors include duraplasty details, operative time, and size of the defect, along with many other preoperative, perioperative, and postoperative variables. Therefore, a large institutional randomized clinical trial including further clinical details would provide insight into this very important topic. However, our data suggests that when choosing a PEEK implant, its characteristics may be important in reducing complication rates. With cranioplasties being one of the most common neurosurgical procedures, it is important to invest time and resources to reduce the large complication rate.

## Conclusion

Our retrospective analysis of 94 patients undergoing cranioplasty with smooth versus perforated PEEK implants revealed no statistically significant differences in complication rates or clinical outcomes between the two implant types. However, trends suggested potential increases in wound complications, postoperative infections, and ventriculoperitoneal shunt placements associated with smooth implants, warranting further investigation.

Given the high prevalence of cranioplasty procedures and the associated complication rates, these findings support continued investigation into the role of implant surface characteristics and their impact on patient outcomes. Future prospective randomized studies are essential to clarify these preliminary trends and optimize implant selection strategies to enhance patient safety and clinical efficacy.

## Data Availability

Due to our study population size and the unique variables we were interested in, our data is not publicly available/posted anywhere. However, we are happy to provide it to the journal and anyone else at their request.

## Abbreviations

CT: Computed tomography
ICH: Intracranial hemorrhage
IQR: Interquartile range
MCE: Malignant cerebral edema
MRI: Magnetic resonance imaging
PEEK: Polyetheretherketone
PMMA: Polymethyl methacrylate
TM: Titanium mesh
VP: Ventriculoperitoneal
